# Epstein-Barr Virus-Positive Lymphomas Exploit Ectonucleotidase Activity To Limit Immune Responses and Prevent Cell Death

**DOI:** 10.1128/mbio.03459-22

**Published:** 2023-02-14

**Authors:** Philip T. Lange, Blossom Damania

**Affiliations:** a Lineberger Comprehensive Cancer Center, The University of North Carolina at Chapel Hill, Chapel Hill, North Carolina, USA; Icahn School of Medicine at Mount Sinai

**Keywords:** EBV, ectonucleotidase, immune evasion, lymphoma

## Abstract

Epstein-Barr virus (EBV) is a cancer-associated virus that infects more than 90% of adults. Unfortunately, many EBV-driven malignancies, including numerous B cell lymphomas, are highly aggressive and lack acceptable therapeutic outcomes. The concentrations of extracellular purines, namely, ATP and adenosine, are highly dysregulated in the tumor microenvironment and significantly impact the degree of immune responses to the tumor. Additionally, many tumor cells adapt to this dysregulation by overexpressing one or more ectonucleotidases, enzymes that degrade extracellular nucleotides to nucleosides. The degradation of immunostimulatory extracellular ATP to immunosuppressive adenosine through ectonucleotidase activity is one example of tumor cell exploitation of the purinergic signaling pathway. As such, preclinical studies targeting the purinergic signaling pathway have found it to be a promising immunotherapeutic target for the treatment of solid tumors; however, the extent to which purinergic signaling impacts the development and survival of EBV^+^ B cell lymphoma remains unstudied. Here, we demonstrate robust ectonucleotidase expression on multiple types of EBV-positive B cell non-Hodgkin lymphoma (NHL). Furthermore, the presence of high concentrations of extracellular ATP resulted in the expression of lytic viral proteins and exhibited cytotoxicity toward EBV^+^ B cell lines, particularly when CD39 was inhibited. Inhibition of CD39 also significantly prolonged survival in an aggressive cord blood humanized mouse model of EBV-driven lymphomagenesis and was correlated with an enhanced inflammatory immune response and reduced tumor burden. Taken together, these data suggest that EBV^+^ B cell lymphomas exploit ectonucleotidase activity to circumvent ATP-mediated inflammation and cell death.

## INTRODUCTION

Gammaherpesviruses are ubiquitous pathogens that establish lifelong infections. The human gammaherpesvirus, Epstein-Barr virus (EBV), infects greater than 90% of the adult population and is associated with numerous malignancies, including gastric cancer, nasopharyngeal carcinoma, and numerous subtypes of non-Hodgkin lymphoma (NHL) including B, T, and NK cell lymphomas, with B cell lymphomas contributing the greatest cancer burden of EBV-associated malignancies. Thus, novel therapeutic approaches for the treatment of EBV-associated lymphomas are desperately needed.

The purinergic signaling pathway is defined by signaling through extracellular purines, namely, ATP and adenosine, and represents a promising clinical target for the treatment of cancer, infectious disease, and autoimmune disorders (1, 2). Extracellular ATP has long been appreciated to mediate inflammatory effects and to be required for robust immune cell activation (3–5). Conversely, adenosine signaling generally dampens inflammatory immune responses. Adenosinergic signaling inhibits B and T cell receptor activation and inflammatory cytokine production by macrophages, NK cells, and dendritic cells, as well as phagocytosis by macrophages (6–10). Furthermore, adenosine promotes regulatory and/or tolerogenic phenotypes in multiple immune cell types (11, 12).

As extracellular purines play such a critical role in the determination of immune responses, their concentrations are tightly controlled via enzymes called ectonucleotidases, ectoenzymes that sequentially dephosphorylate extracellular nucleotides. The two best-studied ectonucleotidases are CD39, which degrades extracellular ATP and ADP to AMP, and CD73, which degrades AMP to adenosine. Thus, ectonucleotidase activity limits inflammatory signaling by degrading ATP, while simultaneously promoting a local immunosuppressive environment through the generation of adenosine.

The extracellular concentration of ATP in healthy tissue is generally very low (1 to 100 nM) but can be increased under various conditions, such as inflammation, hypoxia, and cancer (13–15). Furthermore, it is now thought that extracellular ATP concentrations in the immediate pericellular region are considerably higher than what has been reported via assays that measure ATP in a bulk sample (16). Stimuli shown to promote ATP release into the extracellular space include inflammatory signaling (17), hypoxia (18, 19), and chemotherapeutic agents (20). Cell death, in response to these stimuli, or others, is also associated with ATP release (21, 22). Thus, the high degree of cellular stressors and cell death in the tumor microenvironment is associated with a dramatic increase in extracellular ATP relative to healthy tissue. In fact, extracellular ATP concentrations in the tumor microenvironment are capable of reaching high micromolar levels (≥700 μM) (23).

In order to combat the immunostimulatory effects of the elevated extracellular ATP in the tumor microenvironment, tumor cells, and tumor-associated immune cells, can overexpress CD39 and/or CD73 to rapidly convert extracellular ATP into immunosuppressive adenosine (24). Thus, tumor cells exploit ectonucleotidases and adenosinergic signaling to maintain a locally immunosuppressive tumor microenvironment.

Despite this newfound understanding, the degree to which lymphoid malignancies, particularly those that are driven by oncogenic viruses, exploit the activity of ectonucleotidases to circumvent inflammatory immune responses remains unknown. Here, we report high levels of ectonucleotidase expression on multiple types of EBV-positive B cell lymphomas. Furthermore, we ascribe a functional role to these enzymes in limiting the antitumor response both *in vitro* and *in vivo*, as ectonucleotidase inhibition resulted in enhanced cell death and prolonged survival in a humanized mouse model of EBV-driven lymphomagenesis.

## RESULTS

### EBV-positive B cell lines with type III latency exhibit considerable ectonucleotidase expression.

**FIG 1:**
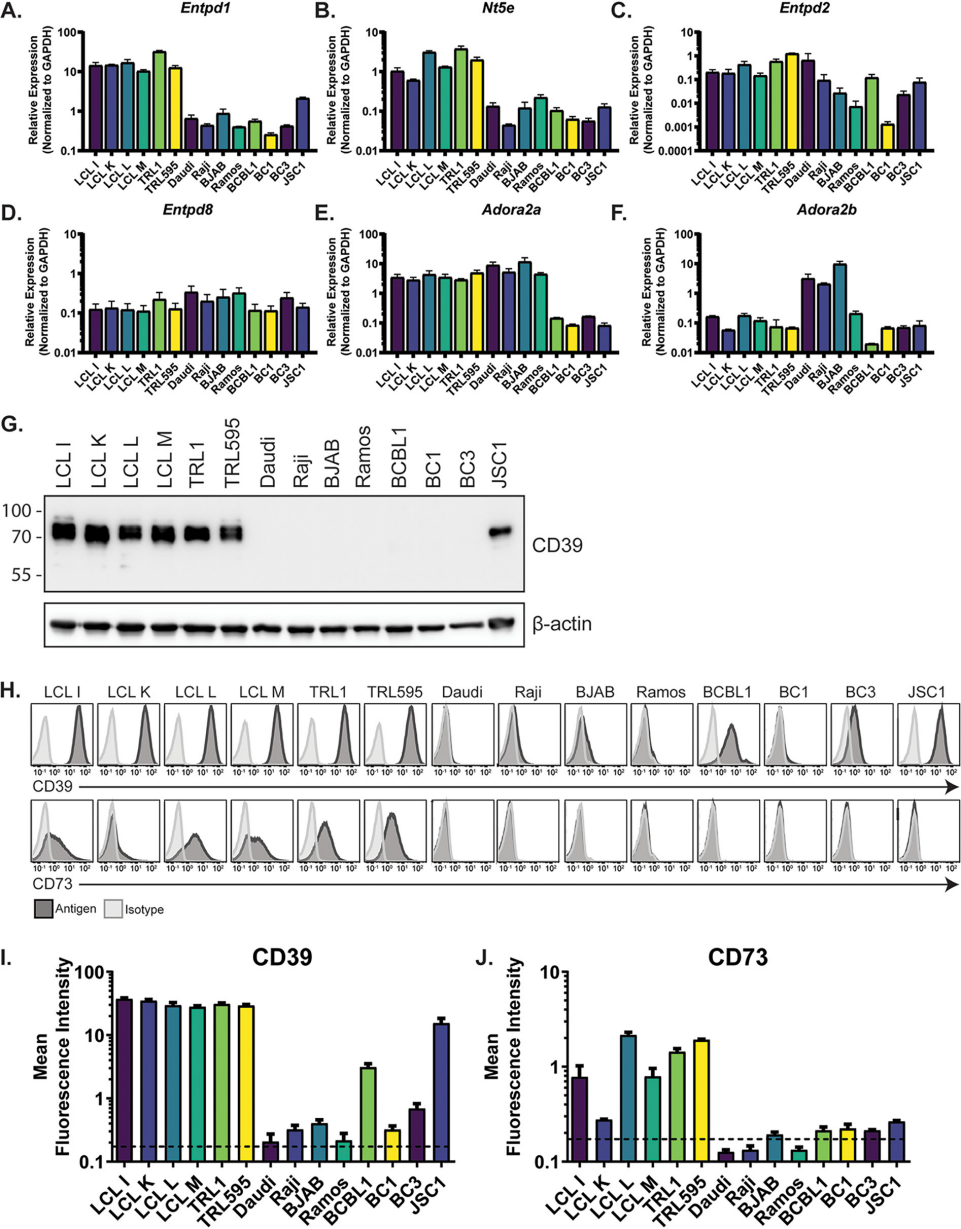
EBV-positive B cells express high levels of ectonucleotidases. (A to F) RNA was isolated from the indicated cell lines, and relative expression of the indicated genes was quantified by qRT-PCR. (G) Protein lysates were collected from the indicated cell lines and probed for relative CD39 and β-actin expression by SDS-PAGE. Numbers at left are molecular masses in kilodaltons. (H to J) Flow cytometric analysis was performed to quantify relative abundance of CD39 and CD73 on the cell surface of the indicated cell lines. Representative FACS histograms are depicted in panels H. I and J display the mean fluorescence intensity pooled from at least three independent biological replicates.

Despite the recent progress in the characterization and therapeutic targeting of ectonucleotidases and purinergic signaling in the context of solid tumors (24), the extent to which purinergic signaling is exploited by viruses and/or lymphoid malignancies remains relatively undescribed. Therefore, in order to characterize the relative expression levels of key players involved in purinergic signaling, bulk RNA was collected from a panel of gammaherpesvirus-associated NHL and EBV-immortalized lymphoblastoid cells. The cell lines examined included four lymphoblastoid cell lines (LCLs; LCL-I, LCL-K, LCL-L, and LCL-M) (25), two B cell lines derived from patients with posttransplant lymphoproliferative disease (PTLD; TRL1 and TRL595) (26, 27), four Burkitt lymphoma cell lines (Daudi, Raji, BJAB, and Ramos), and four primary effusion lymphoma (PEL) cell lines (BCBL1, BC1, BC3, and JSC1) (Table 1). Notably, while all LCLs and PTLD lines are EBV positive, only two of the four Burkitt lymphoma (Daudi and Raji) and two of the four PEL (BC1 and JSC1) lines harbor EBV. Interestingly, the LCLs and PTLD cell lines exhibited significantly higher levels of *Entpd1* (CD39) and *Nt5e* (CD73) transcripts (Fig. 1A and B). The ectonucleotidases *Entpd2* and *Entpd8* were also detected (Fig. 1C and D); however, their expression was generally lower. Furthermore, their pattern of expression did not exhibit any apparent disease-associated differences. The relative expression of adenosine receptors most commonly associated with immune cells (*Adora2a*/A2AR and *Adora2b*/A2BR) was also determined (Fig. 1E and F). Notably, the expression of *Adora2a* was considerably lower in PEL cells than in other cell lines examined, while the expression of *Adora2b* was elevated in most Burkitt lymphoma cell lines.

**TABLE 1:** Details of cell lines used in this study

Cell line	Cell type/derived from	EBV	KSHV^*a*^
LCL-I	Lymphoblastoid cell line	+Latency III	−
LCL-K	Lymphoblastoid cell line	+Latency III	−
LCL-L	Lymphoblastoid cell line	+Latency III	−
LCL-M	Lymphoblastoid cell line	+Latency III	−
TRL1	Posttransplant lymphoproliferative disease	+Latency III	−
TRL595	Posttransplant lymphoproliferative disease	+Latency III	−
Daudi	Burkitt’s lymphoma	+Latency I	−
Raji	Burkitt’s lymphoma	+Latency III	−
BJAB	Burkitt’s lymphoma	−	−
Ramos	Burkitt’s lymphoma	−	−
BCBL1	Primary effusion lymphoma	−	+
BC1	Primary effusion lymphoma	+Latency I	+
BC3	Primary effusion lymphoma	−	+
JSC1	Primary effusion lymphoma	+Latency I	+
GM12878	Lymphoblastoid cell line	+Latency III	−
Akata-BX1	Burkitt’s lymphoma	+Latency I	−

a KSHV, Kaposi’s sarcoma-associated herpesvirus; +, cells are infected with the indicated virus; −, cells are not infected with the indicated virus.

Given the increased transcript levels of CD39 seen in the EBV-positive LCLs and PTLD cell lines, we next examined CD39 protein expression. Relative levels of CD39 protein largely correlated with gene expression, with LCLs and PTLD cell lines showing the greatest expression by Western blotting (Fig. 1G). As CD39 and CD73 are membrane-associated proteins, we also sought to measure their expression at the cell surface via flow cytometry. Concordant with reverse transcriptase quantitative PCR (qRT-PCR) and Western blot analyses, the expression of these enzymes was considerably higher in LCLs and PTLDs than in the other B cell lymphomas examined (Fig. 1H to J). Notably, the LCLs and PTLDs were the only cells that expressed both CD39 and CD73 at levels above background. Furthermore, it is worth noting that these cell lines exhibit type III EBV latency, a viral transcriptional program in which all viral latency genes and noncoding RNAs are expressed. Conversely, EBV-positive Burkitt and PEL cell lines generally display type I latency, which is defined by expression of only EBNA1 and noncoding RNAs, EBV-encoded small RNAs (EBERs), and BamHI-A rightward transcripts (BARTs). Thus, the type of EBV latency associated with each disease correlates with the extent to which the immunosuppressive ectonucleotidases, CD39 and CD73, are expressed.

### LCLs and PTLD lines have high ectonucleotidase activity.

**FIG 2:**
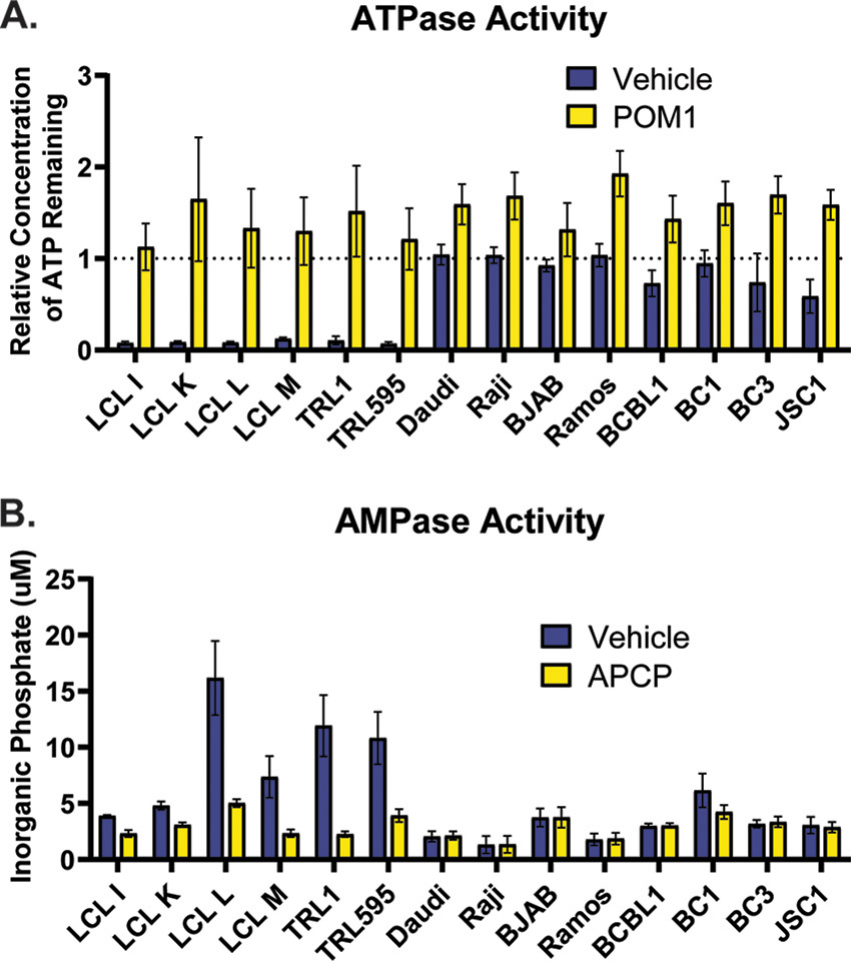
EBV-positive B cells exhibit considerable CD39 and CD73 enzymatic activity. The indicated cell lines were incubated in the presence or absence of the CD39 inhibitor POM1 or the CD73 inhibitor APCP for 15 min at 37°C. A 100 μM bolus of ATP (A) or 10 μM bolus of AMP (B) was added to the culture, and cells were incubated at 37°C. (A) CD39 activity was assessed by determining the relative concentration of ATP remaining in the culture following a 15-min incubation at 37°C. (B) CD73 activity was assessed by determining the concentration of inorganic phosphate released into the medium following a 1-h incubation at 37°C. Data are pooled from three or more independent experiments.

Having observed robust ectonucleotidase expression in LCLs and PTLD cell lines, we next sought to quantify the relative CD39 and CD73 enzymatic activity across the same panel of B cell lines. To compare CD39 activities, a bolus of ATP was added to each cell culture and the concentration of ATP remaining after 15 min was determined (Fig. 2A). Remarkably, all of the LCL and PTLD lines showed nearly complete degradation of the added ATP following the 15-minute incubation, concordant with the observed expression pattern of CD39 in these cell lines. On the other hand, the Burkitt and PEL lines exhibited comparatively little ATPase activity. The ATPase activity measured was driven by CD39, as pretreating the cells with the small-molecule CD39 inhibitor, POM1, completely abrogated the degradation of ATP (Fig. 2A).

CD73 enzymatic activities were compared similarly; however, rather than measuring the remaining AMP in the culture, the accumulation of inorganic phosphate was determined as a proxy for AMP degradation (Fig. 2B). The production of inorganic phosphate was most apparent in the LCL-L, TRL1, and TRL595 cultures and appeared to be CD73 dependent, as pretreatment with an inhibitor of CD73 [adenosine 5′-(α,β-methylene)diphosphate (APCP)] abolished this activity. Thus, LCLs and PTLD lines exhibit considerable CD39-mediated ATPase activity and, in many cases, measurable CD73-mediated AMPase activity.

### High concentrations of extracellular ATP drive EBV reactivation and cell death.

**FIG 3:**
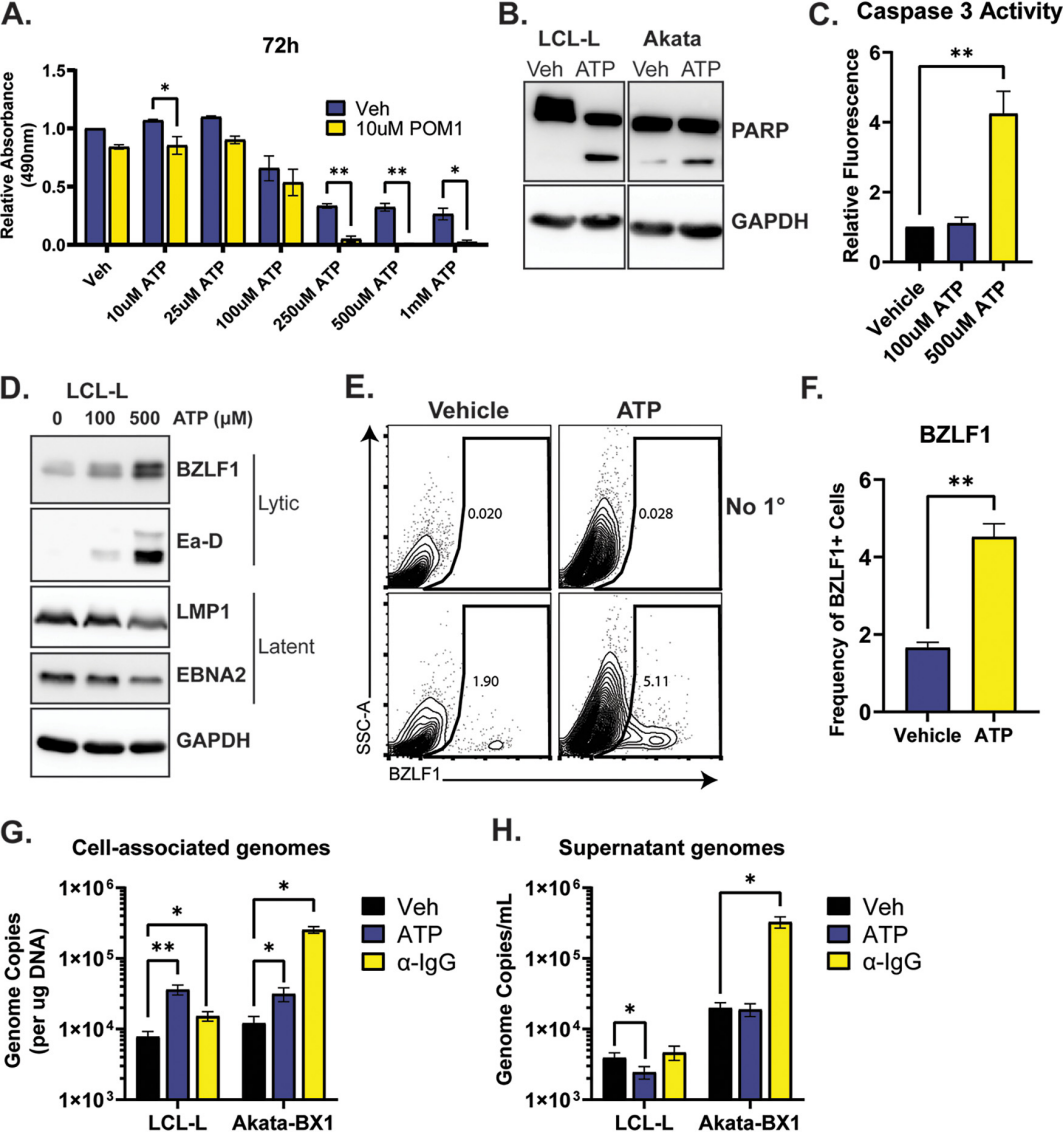
High concentrations of extracellular ATP drive EBV reactivation and cell death. (A) LCLs were incubated with or without POM1, after which the indicated concentrations of ATP were added. ATP was replenished daily. At the indicated time points, relative cell densities were determined by MTS assay. (B) LCL-L or Akata-BX1 cells were treated with 500 μM ATP daily for 3 days. Protein lysates were collected and subjected to SDS-PAGE analysis for the indicated proteins. (C) LCL-L cells were treated with the indicated concentrations of ATP daily. At 48 h posttreatment, the cells were collected and caspase-3 activity was determined via ApoAlert caspase-3 fluorescent assay kit. (D) LCL-L cells were treated with the indicated concentrations of ATP daily. At 72 h posttreatment, cell lysates were collected and subjected to SDS-PAGE analysis for the indicated proteins. (E and F) LCL-L cells were treated with 500 μM ATP or vehicle control daily. At 48 h posttreatment, the cells were stained for intracellular BZLF1 and analyzed by flow cytometry. Panel E depicts a representative flow cytometry panel and gating, while panel F is the quantitation from three independent experiments. SSC, side scatter. (G and H) The indicated cell lines were treated with 500 μM ATP daily or 25 μg/mL goat anti-human IgG. At 72 h posttreatment, total DNA was isolated from pelleted cells (G) and supernatant (H). The number of EBV genome copies in each fraction was determined by qPCR using a standard curve derived with a BMRF1-encoding plasmid. Data in panels B, D, and E are representative of at least three independent experiments. Data in other panels are pooled from at least three independent experiments.

Extracellular ATP is generally maintained at concentrations much lower than the intracellular concentrations; however, levels of extracellular ATP in the tumor microenvironment have been reported to reach concentrations upward of 700 μM (23). Thus, we next sought to investigate how the dynamic changes in extracellular ATP that occur in the tumor microenvironment impact EBV-positive B cell biology. First, LCL proliferation and viability were quantified following treatment with increasing concentrations of ATP. The relative number of viable cells decreased with increasing extracellular ATP concentrations (Fig. 3A). Notably, pretreatment of the LCLs with a low concentration of POM1 further sensitized the cells to the cytotoxic effects of ATP, particularly at high concentrations of ATP. Furthermore, both LCLs (type III latency) and Akata cells (Burkitt lymphoma, type I latency) displayed evidence of apoptosis following treatment with ATP or POM1, as indicated by the accumulation of cleaved poly(ADP-ribose) polymerase (PARP) (Fig. 3B; see also Fig. S1A in the supplemental material). In order to confirm activation of apoptotic cell death, LCLs were treated with ATP or POM1 and caspase-3 activity was quantified. Indeed, caspase-3 activity was elevated in a dose-dependent manner following treatment with either molecule, with 500 μM ATP driving a nearly 5-fold increase in caspase-3 activity (Fig. 3C; Fig. S1B). Interestingly, the ATP-driven cell death correlated with a substantial increase in EBV lytic protein expression (Fig. 3D to F). Notably, treatment with the CD73 inhibitor APCP did not result in cell death, nor the expression of lytic viral proteins (Fig. S1A and B).

10.1128/mbio.03459-22.1 FIG S1 Activating purinergic signaling drives abortive lytic reactivation and apoptotic cell death. (A and B) LCLs were treated with the indicated concentrations of POM1 or APCP. At 48 h posttreatment, protein lysates were collected and subjected to SDS-PAGE analysis for the indicated proteins (A), or lysates were collected and relative caspase-3 activity was determined via ApoAlert caspase-3 fluorescent assay kit. Panel A is representative of three independent experiments, and panel B is pooled data from three independent experiments. (C to E) LCLs and Akata-BX1 cells were treated with the indicated concentrations of ATP, 25 μg/mL goat anti-human IgG, or vehicle control. At 48 and 72 h posttreatment, cells were collected and expression of gp350 was determined by flow cytometry. Panel C shows representative flow plots of LCLs at 48 h posttreatment. Panels D and E show the quantification of three independent experiments. Download FIG S1, PDF file, 0.4 MB. Copyright © 2023 Lange and Damania. 2023 Lange and Damania. https://creativecommons.org/licenses/by/4.0/ This content is distributed under the terms of the Creative Commons Attribution 4.0 International license.

Having observed this dramatic induction of EBV lytic protein expression following ATP and POM1 treatment, we wanted to ascertain if this was indicative of productive viral reactivation and the release of progeny virions. Quantification of cell-associated EBV genomes showed a significant increase in ATP-treated LCLs and Akata cells when normalized to total DNA content (Fig. 3G). Surprisingly, despite the increase in cell-associated genomes, no increase was detected in the concentration of DNase-resistant viral genomes in the supernatant (Fig. 3H). Additionally, expression of the viral glycoprotein gp350 was not induced by treatment with ATP (Fig. S1C to S1E). Thus, although high concentrations of extracellular ATP drive cell death and abortive viral lytic gene expression, infectious virions are not produced.

### CD39 inhibition prolongs survival and enhances immune responses in a cord blood humanized mouse model of EBV-driven lymphomagenesis.

**FIG 4:**
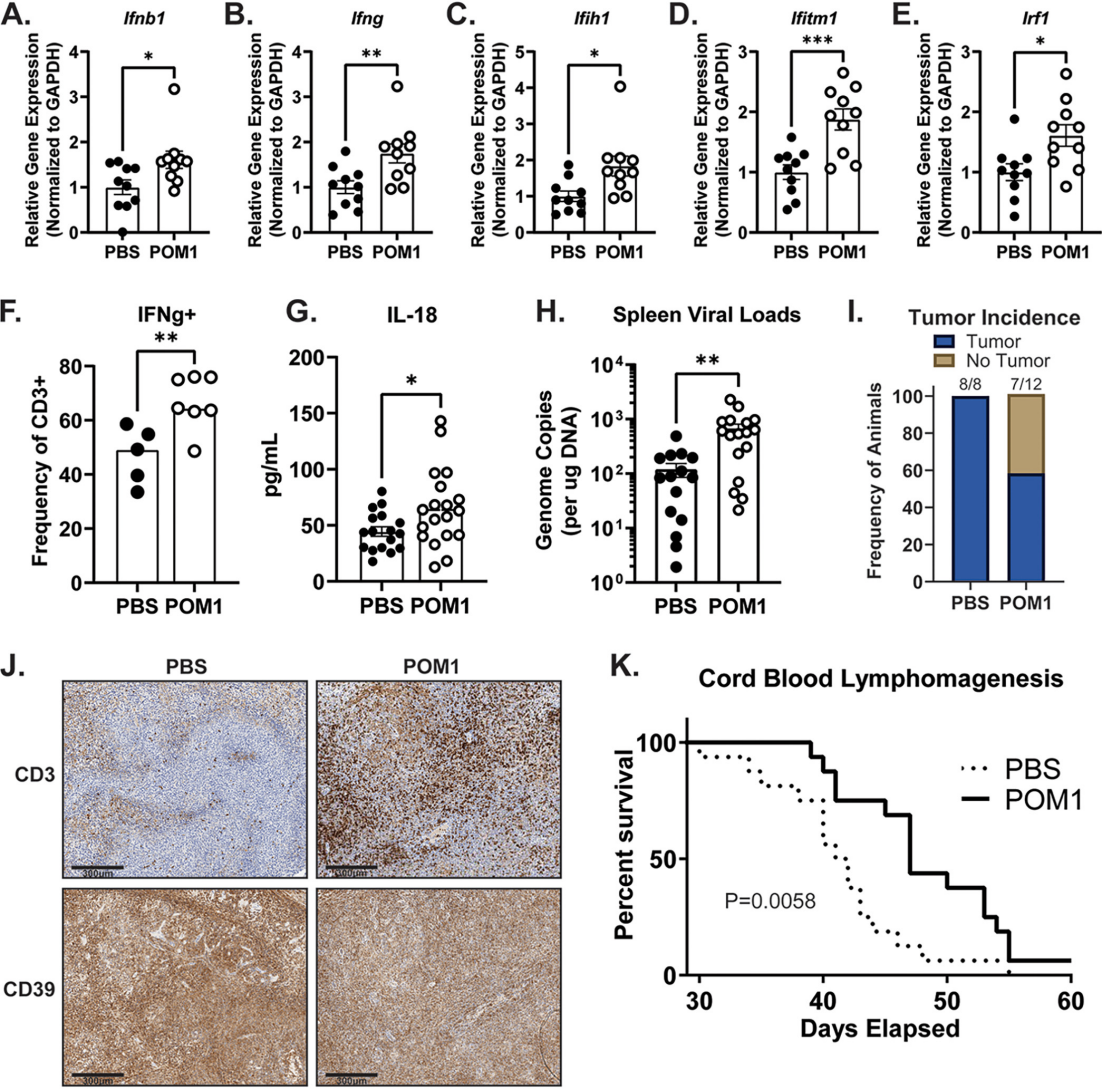
CD39 inhibition correlates with enhanced inflammatory responses and prolonged survival in a cord blood humanized mouse model of EBV-driven lymphomagenesis. Human cord blood mononuclear cells were infected with the Akata-BX1 strain of EBV for 1 to 1.5 h at 37°C. The infected cord blood cells were then intraperitoneally injected into 3- to 5-week-old female NSG mice. Five days postinjection, mice were randomized into treatment groups and began receiving POM1 (5 mg/kg) or PBS injections intraperitoneally 5 days per week. (A to J) At 28 days postinjection, mice were euthanized, and spleens, tumors, and sera were collected for analysis. (A to E) Splenic tissue was homogenized in TRIzol, and bulk RNA was isolated. The relative expression of the indicated genes was determined by qRT-PCR. (F) Spleens were processed into single cell suspensions, restimulated with phorbol myristate acetate (PMA) and ionomycin, and then stained for intracellular IFN-γ and analyzed by flow cytometry. (G) Mouse serum was subjected to Luminex analysis for IL-18. (H) DNA was isolated from splenic tissue homogenized in panels A to E. EBV genome copies were determined via qPCR using a standard curve of a BMRF1-encoding plasmid. (I) At the time of necropsy (28 days), mice were examined for any observable tumors. (J) Tumor tissue was subjected to immunohistochemical staining for CD3 and CD39. Representative images are shown. (K) Mice were infected and treated as described above and euthanized when humane endpoints were reached. Where applicable, data are pooled from 2 to 4 independent experiments with unique cord blood donors.

EBV exhibits exquisite tropism for human cells. As such, animal models of EBV infection are limited to related rodent gammaherpesviruses or the use of humanized mice (28–30). One such humanized mouse model involves the injection of EBV-infected human cord blood mononuclear cells into NOD/LtSz-*scid*/*IL2Rγ^null^* (NSG) mice. The EBV-infected animals develop aggressive B cell lymphomas with type III viral latency resembling human activated B cell-like diffuse large B cell lymphoma and reach humane endpoints within 1 to 2 months with nearly 100% penetrance (28). Furthermore, the presence of EBV allows the human B cells to persist *in vivo* for the duration of the experiment. Human T cells persist regardless of EBV infection and show antitumor activity, tumor infiltration, and responsiveness to immunotherapeutic treatments (28, 31). Thus, we employed this robust lymphomagenesis model in order to determine the extent to which ectonucleotidase activity contributes to the development of, and the immune response to, EBV-positive B cell lymphomas *in vivo*. Briefly, human cord blood mononuclear cells from healthy donors were infected with the Akata-BX1 strain of EBV and then intraperitoneally injected into 3- to 5-week-old female NSG mice. At 5 days postinfection, the mice began receiving injections of the CD39 inhibitor POM1. All mice were euthanized at 28 days postinfection, at which point spleens, sera, and any observable tumors were collected for immunological analyses. Examination of bulk RNA from the spleens of infected animals showed that POM1-treated mice had elevated levels of cellular antiviral gene transcripts (Fig. 4A to E). Additionally, T cells from POM1-treated animals had a greater capacity to produce interferon gamma (IFN-γ) when restimulated *ex vivo* (Fig. 4F). Thus, POM1 treatment correlated with elevated expression of antiviral and antitumor factors by immune cells in the spleen. Consistent with this heightened immune response in the spleen, POM1-treated animals also exhibited a significant increase in systemic levels of the inflammatory cytokine, interleukin-18 (IL-18), compared to control animals (Fig. 4G). Thus, CD39 inhibition is correlated with an increased inflammatory state, which would be hypothesized to allow for an enhanced antiviral and antitumor response. Interestingly, as was observed in ATP-treated cells *in vitro*, CD39 inhibition was correlated with a significant increase in cell-associated EBV genomes in the spleens of cord blood humanized mice (Fig. 4H). Remarkably, tumor incidence at 28 days postinfection was strikingly different between the two cohorts. While every phosphate-buffered saline (PBS)-treated animal had obvious tumors upon necroscopic examination, only some of the POM1-treated animals had identifiable tumors (Fig. 4I). POM1-treated animals that did develop tumors by 28 days postinfection showed an increased level of T cell infiltration into the tumor tissue (Fig. 4J), suggesting that the increase in inflammatory mediators observed in the spleen and serum of POM1-treated mice is also correlated with an enhanced antitumor immune response at the site of lymphomagenesis. Expectedly, histological staining also confirmed high expression of CD39 on tumor cells (Fig. 4J).

Finally, we sought to determine the extent to which CD39 inhibition altered survival in this aggressive model of EBV-driven lymphomagenesis. Mice were injected with EBV-infected cord blood mononuclear cells and treated with POM1 as described above. Treatments continued 5 days per week until humane endpoints were reached. Remarkably, the resulting data demonstrate that monotherapeutic administration of POM1 significantly prolongs survival in this model of aggressive EBV-driven lymphomagenesis (Fig. 4K).

## DISCUSSION

**FIG 5:**
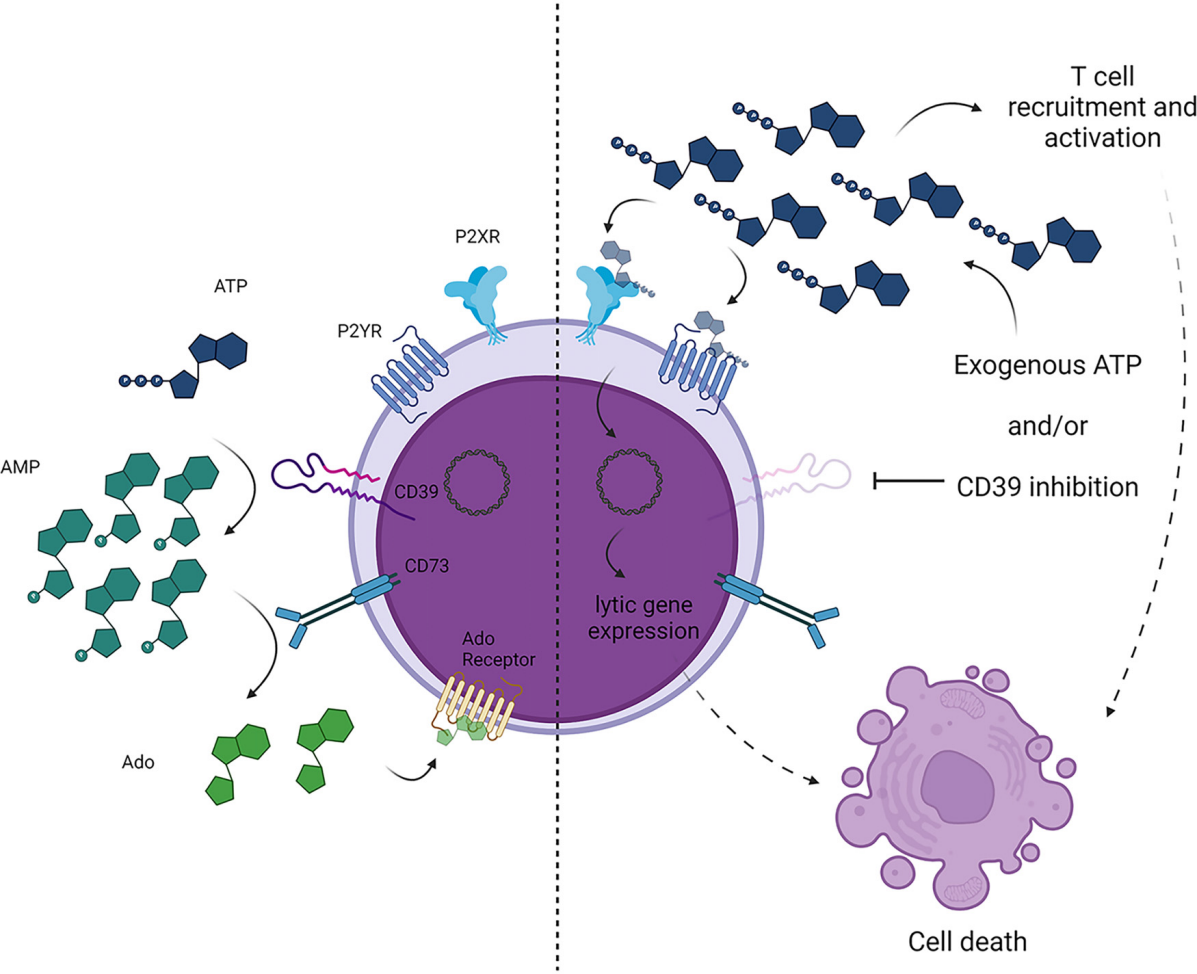
Working model. Under steady-state conditions (left), EBV^+^ B cell lymphomas express high levels of ectonucleotidases to rapidly degrade immunostimulatory extracellular ATP to immunosuppressive adenosine. This limits ATP-mediated P2 receptor signaling and subsequent lytic gene expression and promotes a locally immunosuppressive environment to restrict inflammatory and/or cytotoxic immune responses to the lymphoma cells. When CD39 activity is inhibited and/or extracellular ATP levels are significantly elevated, the rise in extracellular ATP promotes a locally immunostimulatory environment and lytic viral gene expression, allowing for more robust antitumor immune responses.

To our knowledge, this study is the first to thoroughly examine the extent to which an oncogenic virus and lymphoid malignancies exploit extracellular purine catabolism and purinergic signaling. Here, we demonstrate that a subset of EBV-driven B cell malignancies express high levels of ectonucleotidases, particularly CD39. The robust CD39 expression correlates with considerable ATPase activity of LCLs and PTLD-derived B cell lines. Additionally, while the expression of CD73 appears to be markedly lower than that of CD39, these cells also display measurable CD73-dependent AMPase activity. Together, these data suggest that certain EBV-positive lymphoid malignancies have the capacity to deplete the surrounding environment of immunostimulatory extracellular ATP, generating immunosuppressive extracellular adenosine in the process. Interestingly, while EBV^+^ LCLs and PTLD cells show robust expression of CD39 and measurable expression of CD73, EBV^+^ PEL (BC1 and JSC1) and EBV^+^ Burkitt lymphoma (Daudi and Raji) cell lines exhibit little to no detectable ectonucleotidase expression. Unlike LCLs and PTLD cells, which display latency III gene expression, PEL and Burkitt lymphoma cells typically exhibit type I latency. As such, it is tempting to speculate on a role for EBV latency II- or III-restricted genes in driving ectonucleotidase expression, possibly as a means to temper the immune response to these more immunogenic gene expression programs. In addition to the role of ectonucleotidase activity in preventing immune cell activation, the degradation of extracellular ATP may be a means by which EBV is able to maintain viral latency in transformed cells, as we have found that high levels of extracellular ATP promote abortive reactivation with the expression of lytic proteins and are associated with cell death (Fig. 5). Inhibition of CD39 prolongs survival in a humanized mouse model of EBV-driven lymphomagenesis, with POM1-treated animals also showing an enhanced inflammatory signature and increased T cell infiltration into the tumors. Remarkably, while all vehicle-treated animals displayed grossly observable tumors by day 28 postinfection, only some of the POM1-treated animals showed evidence of lymphomagenesis. Thus, CD39 represents a promising therapeutic target for the management of EBV-driven B cell malignancies.

### Purinergic signaling and lytic induction therapy.

Lytic induction therapy is a potential strategy to treat EBV-positive malignancies. Traditionally, lytic induction therapy involves a stimulus that promotes viral reactivation from latency (e.g., histone deacetylase [HDAC] inhibitors, phorbol esters, butyrates, etc.) followed by treatment with a nucleoside analog, such as ganciclovir. The ganciclovir prodrug is then converted to its cytotoxic form by the viral protein kinase BGLF4, subsequently killing the reactivating cells as well as bystander cells (32). Recently, a related approach has been proposed in which tumors exhibiting the sparingly immunogenic viral latency program, latency I, are driven into more immunogenic latency II or III transcriptional programs, making the tumor cells a target for EBV-specific cytotoxic T lymphocytes (CTLs) that recognize antigens of other latency proteins (e.g., latent membrane protein 1 [LMP1], EBNA3, etc.) (33). The referenced study utilized the hypomethylating compound decitabine to convert EBV-infected B cell lines from latency I to latency II or III, with *in vitro* latency program conversion reaching ~60% under some conditions.

Importantly, there are some caveats to lytic induction therapy and epigenetic latency reprogramming. Lytic induction therapy may induce adverse events due to overly robust reactivation and associated viremia (34). As many EBV latency genes are oncogenic, reprogramming cells to a more immunogenic latency type may also promote a greater rate of proliferation and increased resistance to cell death. Notably, combinatorial strategies may be developed by driving lytic reactivation and then adoptively transferring CTLs targeting lytic antigens, with the antiviral ganciclovir, to prevent the production of infectious EBV following reactivation. Furthermore, due to the risk of viremia that is associated with lytic induction therapy, compounds that induce lytic gene expression but not the production of progeny virions may be particularly desirable.

We found that ATP stimulation promoted viral genome replication in both latency I and latency III cells. Notably, while cell-associated EBV genomes were increased by ATP, there was no detectable increase in DNase-resistant cell-free genomes (Fig. 3G and H), suggesting that ATP treatment may promote an abortive lytic cycle. Furthermore, while the early lytic proteins Zta and Ea-D were robustly induced by ATP in a dose-dependent manner (Fig. 3D to F), we were unable to detect induction of the late protein gp350 (see Fig. S1C to E in the supplemental material), further suggesting that ATP does not drive infectious virion production. Thus, purinergic signaling may represent an attractive combinatorial target for lytic induction therapy.

### Purinergic signaling and the host immune response.

The purinergic signaling pathway has garnered significant attention in recent years for its critical role in determining the type and quality of immune response to tumors. Here, we demonstrate high levels of ectonucleotidases in EBV-positive B cells, particularly those exhibiting the type III latency program. The correlation between ectonucleotidase expression and the immunogenic latency III gene expression program suggests that EBV-transformed B cells may elevate expression of these enzymes in order to counteract the immune response in the face of elevated viral immunogenicity. Notably, expression of one viral oncoprotein, latent membrane protein 1 (LMP1), was shown to stimulate CD39 expression in some cell lines (35). Further, knockdown of LMP1 resulted in a dramatic reduction in CD39 protein expression (36). Thus, it is possible that the abundant CD39 expression observed in LCLs and PTLD lines may be driven by LMP1, in which case cells with latency II viral gene expression would also be expected to exhibit high CD39 expression and activity.

In our studies of EBV-driven lymphomagenesis, POM1-treated animals displayed a more robust immune response than control-treated mice. Bulk splenocyte gene expression analysis revealed significantly higher levels of type I and II interferons and multiple interferon-stimulated genes. Furthermore, mice in which CD39 was inhibited had a greater number of T cells in the spleen capable of producing IFN-γ, as well as more T cells infiltrating the solid tumor masses. These data suggest that at least some EBV-positive B cell lymphomas utilize CD39 activity to hinder innate and adaptive immune responses to the virus and/or malignant cells.

Notably, in addition to more robust antiviral and antitumor immune responses, POM1-treated animals had more EBV genome copies in the spleen at 28 days postinfection. As CD39 inhibition is expected to increase local extracellular ATP concentrations, this finding likely mirrors the increase in cell-associated genomes that we observed following ATP treatment *in vitro* (Fig. 3). Thus, CD39 inhibition likely enhances antigenicity of EBV-infected cells without the risks associated with uncontrolled viremia. Preclinical studies targeting CD39 and/or CD73 in animal models of solid tumors have demonstrated promising results (37–40). In summary, we demonstrate for the first time the value of targeting the purinergic signaling axis in EBV-driven lymphomagenesis. Our findings support the feasibility of immunotherapeutic approaches for the treatment of viral lymphomas and lend credence to the purinergic signaling pathway as a viable therapeutic target for these malignancies.

## MATERIALS AND METHODS

### Cell culture.

All cell lines were grown at 37°C with 5% CO_2_. All B cell lines, except for primary effusion lymphoma and Akata-BX1 cells, were grown in RPMI 1640 medium supplemented with 10% fetal bovine serum (FBS), 1% penicillin-streptomycin, and 1% l-glutamine. Primary effusion lymphoma cell lines were grown in the same complete RPMI medium as described above further supplemented with 0.075% sodium bicarbonate and 0.05 mM β-mercaptoethanol. Akata-BX1 cells (a kind gift from Lindsey Hutt-Fletcher) were cultured in the same base RPMI medium supplemented with 500 μg/mL Geneticin. The TRL1 and TRL595 cell lines were a kind gift from Dr. Cliona Rooney.

### Chemicals and reagents.

Sodium metatungstate (POM1; 21160) and AMP (catalog no. 21094) were purchased from Cayman Chemicals. ATP was purchased from Sigma-Aldrich (catalog no. A1852). Adenosine 5′-(α,β-methylene)diphosphate (APCP) was purchased from Sigma-Aldrich (catalog no. M3763).

### Immunoblotting.

Cells were pelleted and lysed in radioimmunoprecipitation assay (RIPA) buffer (50 mM Tris-HCl [pH 8], 150 mM NaCl, 1% NP-40, 0.1% SDS, 0.5% sodium deoxycholate) containing 1× Roche protease inhibitor tablet (Sigma-Aldrich; 11836145001) and incubated on ice for 20 to 30 min. Lysates were then spun at 20,000 × *g* for 10 min at 4°C, and the clarified lysates were transferred to clean tubes. Protein concentrations were determined via bicinchoninic acid (BCA) assay (Thermo Scientific; 23227). Protein lysates were denatured and solubilized with 5× urea buffer (10% [wt/vol] SDS, 10 mM β-mercaptoethanol, 20% [vol/vol] glycerol, 0.2 M Tris-HCl [pH 6.8], 0.05% [wt/vol] bromophenol blue, and 8 M urea), and between 20 and 50 μg of protein was loaded per lane of an acrylamide gel. Proteins were resolved by SDS-PAGE and then transferred onto a nitrocellulose membrane. Blots were probed with the following primary antibodies: CD39 (1:1,000; Abcam; ab108248), β-actin–horseradish peroxidase (HRP) (1:1,000; Santa Cruz; sc-47778), PARP (1:1,000; Cell Signaling Technologies; 9542S), glyceraldehyde-3-phosphate dehydrogenase (GAPDH) (1:1,000; Santa Cruz; sc-47724), BZLF1 (1:1,000; Santa Cruz; sc-53904), Ea-D (1:1,000; Santa Cruz; sc-58121), LMP1 (1:1,000; Abcam; ab78113), and EBNA2 (1:1,000; Abcam; ab90543). HRP-linked anti-mouse IgG (1:2,500; Cell Signaling Technologies; 7076S) and HRP-linked anti-rabbit IgG (1:2,500; Cell Signaling Technologies; 7074S) were used for detection of the primary antibodies. Blots were developed with Clarity Western enhanced chemiluminescence (ECL) substrate (Bio-Rad; 1705060) or ECL Prime (GE Healthcare; RPN2232). Blots were imaged using a ChemiDoc imaging system and viewed with Image Lab v5.2.1 software.

### Flow cytometry.

Cells were pelleted and washed once with PBS. Cells were then resuspended at 1 × 10^7^ cells/mL and incubated with Human TruStain FcX (BioLegend; 422302) for 5 min at room temperature. Cells were pelleted and resuspended as described above and then incubated with the following primary antibodies for 30 min on ice: CD39-phycoerythrin (PE) (1:400; BioLegend; 328208), CD73-PE (1:400; BioLegend; 344004), CD3-Alexa Fluor 594 (1:200; BioLegend; 300446), CD4-Pacific Blue (1:400; BioLegend; 344620), CD8-Alexa Fluor 700 (1:400; BioLegend; 300920). To detect cell surface gp350 expression, cells were first incubated with or without unconjugated gp350 antibody (Santa Cruz; sc-57724), washed twice with fluorescence-activated cell sorting (FACS) buffer and then incubated with allophycocyanin (APC)-conjugated goat anti-mouse IgG and washed twice more. To detect intracellular BZLF1 expression, cells were fixed and permeabilized with the True-Nuclear transcription factor buffer set (BioLegend; 424401) according to the manufacturer’s instructions. Cells were first incubated with or without an unconjugated BZLF1 antibody (Santa Cruz; sc-53904), washed twice with permeabilization buffer, and then incubated with APC-conjugated goat anti-mouse IgG and washed twice more. To detect intracellular cytokine expression, cells were fixed and permeabilized with Cyto-Fast Fix/Perm buffer (BioLegend; 426803) according to the manufacturer’s instructions and then incubated with IFN-γ–fluorescein isothiocyanate (FITC) antibody (1:400; BioLegend; 506504) for 20 min at room temperature. In order to gate out dead cells from the analysis, cells were stained with fixable viability dye eFluor 780 (eBioscience; 65-0865-14). Cells were washed with FACS buffer (PBS with 2% FBS, 2 mM EDTA) 2 to 3 times prior to being run on a Becton Dickinson LSRFortessa (for humanized mouse studies) or a Miltenyi MACSQuant VYB (for cell line ectonucleotidase surface expression). Analysis was performed with FlowJo v10.8 software.

### qRT-PCR.

Total RNA was isolated from cell pellets or humanized mouse tissue using TRIzol according to the manufacturer’s instructions, and 2 μg of RNA was DNase treated using the AccuRT genomic DNA removal kit (ABM; G488). The DNase-treated RNA was then split into two 1-μg aliquots; one aliquot was reverse transcribed using the SensiFAST cDNA synthesis kit (Meridian Bioscience; BIO-65054), and the other was treated in the same fashion but without reverse transcriptase (RT). The resulting cDNA (and −RT controls) was diluted 1:4 with water. Real-time PCR was performed using the SensiFAST SYBR Lo-ROX kit (Meridian Bioscience; BIO-94050) on an Applied Biosystems QuantStudio 6 Flex RT-PCR system with the primers shown in Table 2.

**TABLE 2:** Primers for qRT-PCR

Gene	Forward primer (5′–3′)	Reverse primer (5′–3′)
*Entpd1*	GAAGAGCACTGCGCTATAAT	TGCCATGTTGCATCACATA
*Nt5e*	CCTATGCCCAAAGAACAAATAC	GGTGAAGCCTCCCATTTC
*Entpd2*	CTCAATCCAGCTCCTTGAAC	CTACACAGAAGGAGCCTCTAA
*Entpd8*	TGCAAACAGGGCAAGAC	TGCAAACAGGGCAAGAC
*Adora2a*	ACTCTCCCTAGACTCTCCTA	GGGTTTCCTCACACTTACAT
*Adora2b*	CTACACCTCACAAGGAAATGG	GGAGCCTACTACTGACACATA
*Ifnb1*	GTTGAGAACCTCCTGGCTAATG	GGTAATGCAGAATCCTCCCATAATA
*Ifng*	GCATGTCAGACAGAACTTGAATG	GAAGCACCAGGCATGAAATC
*Ifih1*	TGGTGCACAAAGGCTTAGA	GTCAAGATTGGGAAATGTGATAGG
*Ifitm1*	CTGTTACTGGTATTCGGCTCTG	AAAGGTTGCAGGCTATGGG
*Irf1*	GACACAGTCACAGACAGAACA	ACGGTACAGACAGAGCATTTC

### ATPase and AMPase activity assays.

To assess CD39-dependent ATPase activity, cells were first plated in a 48-well plate at a concentration of 4 × 10^5^ cells/mL and incubated with 100 μM POM1 or vehicle control at 37°C for 15 min. ATP was then added to a final concentration of 100 μM, and the plates were returned to a 37°C incubator for 15 min. Following the incubation, 100 μL of medium was collected, immediately placed on ice, and centrifuged at 1,600 × *g* for 3 min at 4°C to remove any cells. The concentration of ATP remaining in the supernatant was then quantified using an ATP assay kit (Abcam; ab83355) and normalized to the concentration of ATP in a well with only medium and ATP.

CD73-dependent AMPase activity was assessed similarly. Briefly, cells were plated at 4 × 10^5^ cells/mL in a 48-well plate and incubated with 100 μM APCP or vehicle control for 15 min at 37°C. AMP was then added to each well to a final concentration of 10 μM, and the plates were returned to a 37°C incubator for 1 h. Following the incubation, 100 μL of medium was collected, immediately placed on ice, and centrifuged at 1,600 × *g* for 3 min at 4°C to remove any cells. As a proxy for AMP degradation, the amount of inorganic phosphate released into the medium was quantified using a malachite green phosphate detection kit (R&D Systems; DY966) according to the manufacturer’s instructions.

### Cell viability assays.

To determine the impact of ATP-mediated signaling on cell proliferation and viability, LCLs were incubated with 10 μM POM1 or vehicle control for 15 min at 37°C, plated in a 96-well plate at 2 × 10^4^ cells/well, treated with the indicated concentrations of ATP, and returned to a 37°C incubator. Due to the rapid degradation of extracellular ATP, ATP was replenished daily. At 72 h posttreatment, the relative concentration of viable cells was determined by 3-(4,5-dimethylthiazol-2-yl)-5-(3-carboxymethoxyphenyl)-2-(4-sulfophenyl)-2H-tetrazolium (MTS) assay (CellTiter 96 AQ_ueous_ One Solution proliferation assay; Promega; G3581) according to the manufacturer’s instructions. Absorbance at 490 nm was quantified using a CLARIOstar Plus microplate reader (BMG Labtech).

### Viral genome quantification.

Cells were plated in 6-well plates at a concentration of 3 × 10^5^ cells/mL and treated with 500 μM ATP, 25 μg/mL goat anti-human IgG, or vehicle control. ATP was replenished daily. At 72 h posttreatment cells were pelleted and total DNA was isolated using a DNeasy blood and tissue kit (Qiagen; 69506). EBV genomes were then quantified from the isolated DNA via quantitative PCR (qPCR) using BMRF1-specific primers (For, CTGAGGAACGAGCAGATGATTG; Rev, CCGGATTGAGTGTCACCTTAAC). Serial dilutions of a BMRF1-containing plasmid were used as a standard curve.

### Cord blood lymphomagenesis model.

Human cord blood mononuclear cells (CBMNCs) were purchased from StemCell Technologies (Vancouver, Canada) and AllCells (Alameda, CA). Unique cord blood donors were used for each individual experiment. CBMNCs were suspended in serum-free RPMI medium (100 million cells/mL) and infected with the Akata-BX1 strain of EBV (2,500 green Raji units per mouse) and incubated at 37°C for 1 to 1.5 h in the presence of DNase (~100 units/mL). Cells were periodically mixed throughout the incubation period to prevent clumping. Following the incubation, cells were replenished with an additional 20 mL of serum-free RPMI medium and centrifuged to remove nonadsorbed virus and DNase. The EBV-infected CBMNCs were resuspended in serum-free RPMI medium and intraperitoneally injected into 3- to 5-week-old female NSG mice at ≥10 million cells per mouse. Mice were treated with 100 μg POM1 (~5 mg/kg of body weight) or PBS 5 days per week beginning 5 days postinfection. Mice were regularly examined for signs of disease development and were euthanized when humane endpoints were reached or when the experiment reached 60 days postinfection. For immunological examination, all experimental and control mice were euthanized at 28 days postinfection, at which time spleens, serum, and any grossly apparent tumors were collected for analysis.

### Processing of spleens and tumors.

Spleens were processed into single cell suspensions for flow cytometric analysis by mechanical disruption and passing through a 100-μm filter. Cell suspensions were then pelleted and treated with red blood cell (RBC) lysis buffer (ACK lysing buffer; Gibco; A1049201) to remove red blood cells. Cells were then replenished with 25 mL of cell medium to dilute the lysis buffer, pelleted by centrifugation, and resuspended in 10 mL of fresh cell medium. Splenocytes were then counted and used for flow cytometric analysis as described above. For gene expression and viral genome quantification, spleens were collected, snap-frozen, and stored at −80°C until processing. Thawed spleens were transferred to screw-cap tubes with 1-mm zirconia beads and 1 mL of TRIzol. Tissues were disrupted using a MagNA Lyser instrument (Roche Diagnostics) at maximum oscillation speed for 30 s, followed by 1 min of cooling on ice, and (if necessary) a second 30-s oscillation time in the MagNA Lyser. The tubes were briefly centrifuged to pellet large debris, and the TRIzol homogenate was transferred to a clean 1.5-mL microcentrifuge tube. Total RNA and DNA were then extracted per the manufacturer’s instructions.

### Luminex analysis.

Serum cytokine analysis was performed using the Cytokine/Chemokine/Growth Factor 45-Plex Human ProcartaPlex panel 1 (Invitrogen; EPX450-12171-901) according to the manufacturer’s instructions. Briefly, magnetic beads were plated in a 96-well plate and washed thoroughly. Serum collected 28 days postinfection was diluted 1:2 with assay buffer, and 50 μL of each sample and standard was added to the plate. The plate was sealed and incubated on a plate shaker at 500 rpm and incubated at room temperature for 30 min. The plate was then transferred to a 4°C refrigerator and incubated overnight. The next day the plate was again placed on a plate shaker at 500 rpm and incubated for 30 min at room temperature. The beads were washed twice, and 25 μL of the detection antibody mixture was added to each well. The plate was incubated at room temperature and washed as described above. After washing, 50 μL of streptavidin-PE was added to each well, and the plate was again incubated for 30 min at room temperature as described above. The plate was washed twice, and the beads were resuspended in 120 μL of reading buffer. Finally, the plate was read using a Luminex Magpix instrument, and the resulting data were analyzed using the ProcartaPlex software (Thermo Fisher).

### Histology.

Tissues collected for histological examination were placed into neutral buffered formalin for fixation. Tissue samples were then submitted to the UNC Pathology Services Core for embedding, sectioning, mounting, immunohistochemical staining, and imaging.

### Caspase-3 activity.

Cells were seeded in six-well plates at a concentration of 5 × 10^5^ cells/mL and treated with the indicated concentrations of ATP or vehicle control for 48 h. As a positive control for caspase-3 activity, one well was treated with 500 nM staurosporine for ~16 h before performing the assay. Caspase-3 activity was then determined using the ApoAlert caspase-3 fluorescent assay kit (TaKaRa Bio; 630217) according to the manufacturer’s instructions.

### Statistical analyses.

All statistical significance was determined using GraphPad Prism 9 software with the indicated statistical analyses: MTS cell viability assays (two-way analysis of variance [ANOVA] with Šídák's multiple comparisons), viral genome quantification (two-tailed, unpaired Student’s *t* test), survival data (log rank [Mantel-Cox] test), gene expression data (two-tailed unpaired Student’s *t* test), BZLF1 flow cytometry data (two-tailed unpaired Student’s *t* test), gp350 flow cytometry (two-way ANOVA with Dunnett’s multiple comparisons), Luminex cytokine analysis (two-tailed unpaired Student’s *t* test). *P* values are denoted throughout the paper as follows: *, *P* ≤ 0.05; **, *P* ≤ 0.01; ***, *P* ≤ 0.001; ****, *P* ≤ 0.0001.
