# Supplementary material for: Epstein-Barr Virus-Positive Lymphomas Exploit Ectonucleotidase Activity To Limit Immune Responses and Prevent Cell Death
Source: mBio. 2023 Feb 14;14(2):e03459-22. doi: 10.1128/mbio.03459-22 (PMC10127690; doi:10.1128/mbio.03459-22)
Supplement: FIG S1 [file mbio.03459-22-s0001.pdf]

Supplemental Material

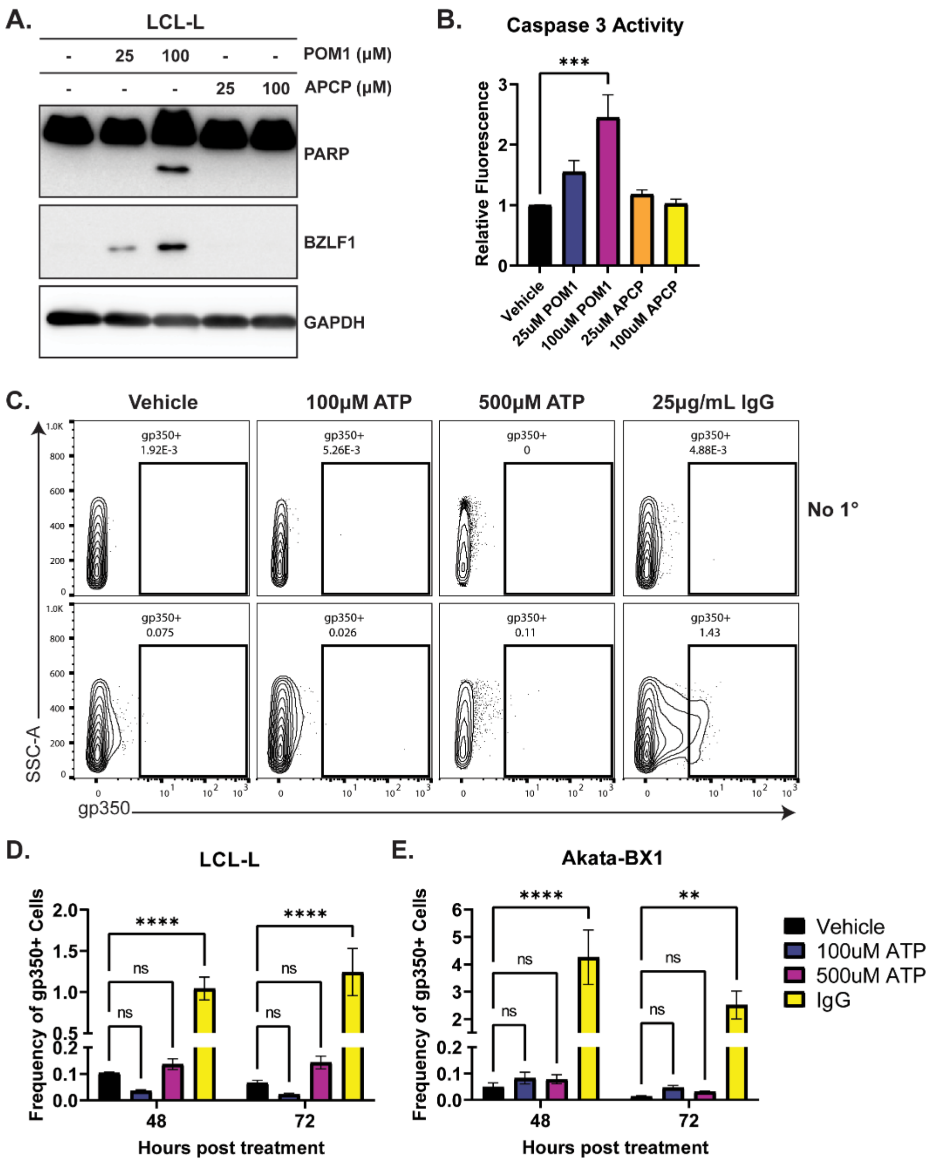

Figure S1. Activating purinergic signaling drives abortive lytic reactivation and apoptotic cell death. A-B) LCLs were treated with the indicated concentrations of POM1 or APCP. At 48 hours post treatment, protein lysates were collected and subjected to SDS-PAGE analysis for the indicated proteins (**A**), or lysates were collected and relative caspase-3 activity was determined via ApoAlert Caspase-3 Fluorescent Assay Kit. Panel (**A**) is representative of three independent experiments and panel (**B**) is pooled data from three independent experiments. **C-E**) LCLs and Akata-BX1 cells were treated with the indicated concentrations of ATP, 25μg/mL goat anti-human IgG, or vehicle control. At 48 and 72 hours post treatment, cells were collected and expression of gp350 was determined by flow cytometry. Panel (**C**) shows representative flow plots of LCLs at 48 hours post treatment. Panels (**D**) and (**E**) show the quantification of three independent experiments.
